# *In vitro* Cytotoxicity Comparison of MTA Fillapex, AH-26 and Apatite Root Canal Sealer at Different Setting Times

**DOI:** 10.22037/iej.2017.32

**Published:** 2017

**Authors:** Farnaz Jafari, Marzieh Aghazadeh, Sanaz Jafari, Faraz Khaki, Fahime Kabiri

**Affiliations:** a *Department of Endodontics, Dental School, Tabriz Branch, Islamic Azad University, Tabriz, Iran; *; b * Oral Health and Community Dentistry Department, Dental School, Tabriz University of Medical Sciences, Tabriz, Iran; *; c * Orthodontics Department, Dental School, Ilam University of Medical Sciences, Ilam, Iran; *; d * Dental School, Tabriz University of Medical Sciences, Tabriz, Iran; *; e *Stem Cell Research Center, Tabriz University of Medical Sciences, Tabriz, Iran*

**Keywords:** Cytotoxicity, MTT Assay, Root Canal Sealer

## Abstract

**Introduction:**

This study aimed to compare the cytotoxicity of MTA Fillapex, AH-26 and Apatite root canal sealers at different times after mixing.

**Methods and Materials:**

In this *in vitro* study, MTA Fillapex, AH-26 and Apatite root canal sealer were spilled uniformly by 40 µm mesh in a 96-well plate. Then, human fetal foreskin fibroblast cell line (HFFF2) were added to each sealer cell culture medium. Cytotoxicity was measured using MTT assay after 24, 48 and 72 h and seven days. Multiple comparisons were done using analysis of variances (ANOVA) and Scheffe’s post hoc test.

**Results:**

All studied sealers exhibited severe cytotoxicity (more than 70%) except for Apatite sealer (95%) at 48 h after mixing. Cytotoxicity of MTA Fillapex and AH-26 were similar (*P*>0.05) at 24, 48 and 72 h and 7 days after mixing of sealers. Cytotoxicity of MTA Fillapex and Apatite root canal sealer, at 24 and 48 h, were significantly different (*P*=0.003 and *P*=0.000, respectively); MTA Fillapex was more cytotoxic. However in 72 h and 7 days after mixing, the difference was not significant (*P*>0.05). At 24 and 48 h after mixing, AH-26 was more cytotoxic (*P*=0.002 and *P*=0.000, respectively). Same as above at 72 h and 7 days after mixing, their cytotoxicity were similar (*P*>0.05).

**Conclusion:**

Overall cytotoxicity of all studied materials were severe. However, it was observed that the cytotoxicity of MTA Fillapex, AH-26 and Apatite root canal sealer decreased over time. Apatite root canal sealer exhibited the least cytotoxicity. Cytotoxicity of MTA Fillapex and AH-26 were similar at different time intervals.

## Introduction

The most commonly used root canal obturation materials are gutta-percha accompanied by endodontic sealers [1]. It is necessary to use sealers to increase the sealing ability of gutta-percha [2]. A hermetic seal after cleaning and shaping of the root canal is a vital factor for the success of endodontic treatment [3]. In addition, sealers serve as a lubricating agent for sliding and easier placement of the main obturating materials and lead to the obturation of accessory canals and fins along the root canal [1]. The sealer and its products within the root canal come into close contact with extracellular fluids and the periapical tissues. Therefore, they might induce various reactions around the root [4-6]. Cytotoxic agents, too, can induce inflammatory reactions and tissue damage [7]. 

MTA Fillapex sealer (Angelus, Londrina, PR, Brazil) is a newly introduced sealer. The philosophy of the production of this sealer is the presence of MTA in its structure, which has various uses in endodontics [8, 9]. One of the characteristics of MTA is its alkaline pH which might make it cytotoxic. However, it exhibits biologic properties and is not cytotoxic after setting [10]. MTA Fillapex also exhibits appropriate antibacterial [11], antifungal [12] and sealing [13] properties.

AH-26 (Dentsply, Tulsa Dental, Tulsa, OK, USA) is a useful and commonly used, but cytotoxic, sealer in root canal therapy; its cytotoxicity is attributed to release of formaldehyde as a result of chemical setting reaction [14-17]. After 24 h, this sealer exhibits minimum toxicity *in vivo* and *in vitro* [17]. 

Apatite root canal sealer (Sankin, Kogyo, Tokyo, Japan) has calcium phosphate and hydroxyapatite in its structure and has been marketed in three generations [18]. The sealer exhibited less bacterial tight seal in comparison with MTA Fillapex and AH-26 sealers [13]. It has antimicrobial properties in its second generation due to the incorporation of 50% iodoform into its structure [18]. Its third generation contains bismuth carbonate, too [18]. The advantage of this sealer is its superb biocompatibility due to the presence of hydroxyapatite [18, 19]. 

The cytotoxicity of sealers is important in relation to their clinical application and no studies are available on the cytotoxic effects of MTA Fillapex, AH-26 and Apatite root canal sealers in the long term subsequent to mixing. The present study was undertaken to evaluate the cytotoxicity of MTA Fillapex, AH-26 and third generation of Apatite root canal sealer at different time intervals after mixing using *in vitro* Mosmann’s Tetrazolium Toxicity (MTT) assay.

## Materials and Methods

MTA Fillapex (Angelus, Londrina, PR, Brazil), AH-26 (Dentsply, Tulsa Dental, Tulsa, OK, USA) and Apatite (Sankin, Kogyo, Tokyo, Japan) sealers were evaluated in the present study.

First, the human fetal foreskin fibroblast cell line (HFFF2), was provided from the Pasteur Institute, Tehran, Iran, and transferred to the laboratory. Then the cells were cultured in flask to reach a level of 2 million cells. To make sure of the number of cells, 1 mL of the flask content was stained with trypan blue and counted using a Neobar lamella. 

Each sealer was added to the floor of the wells using a nylon 40 µm mesh. Then 5000 cells were added to each plate. After 24 h, the adhesion of cells to the floor of the flasks was observed, which made it possible to evaluate the toxicity of each sealer. Cellular treatment was carried out at the relevant time intervals (24, 48 and 72 h and 7 days) and after adding sealer and the appearance of its effects, the color change in the environment was read by microplate reader (ELX808 absorbance microplate reader; BioTek Instruments Inc., Winooski, VT, USA). The procedures were repeated for all the other sealers.

First, AH-26, MTA Fillapex and Apatite root canal sealers were prepared with an appropriate consistency recommended by each manufacturer and sterilized by gamma rays at a dose of 37.2 Gy before adding them to the 96-well culture media. A mesh was used to add 40 µm of sealers to 96-well plate in the culture media. Then fibroblasts were added to wells. Pure fibroblasts were cultured without contacting sealers as negative control group. The cells were allowed 24 h for adhesion. The culture media contained Dulbecco's modified Eagle's medium (DMEM; Gibco Laboratories, Grand Is., NY, USA) with 10% fetal bovine serum (FBS) (PAA, Pasching, Austria) supplemented by penicillin/streptomycin (pen/strep). After 24 h, based on the MTT assay protocol, color changes as a result of cellular death were evaluated through MTT solution (Tetrazelium bromide, Sigma, USA) and DMSO powders. The procedure was repeated with each sealer 8 times in order to achieve more accurate results. A 24-, 48- and 72-h and 7-day intervals, color changes as a result of cellular death were evaluated by MTT assay. It should be pointed out that some relevant wells at each interval were evaluated separately without interference. The cellular culture media were effective for 7 days and did not require any replacement due to the absence of growth and proliferation of fibroblast cells and also due to very limited growth and the enriched nature of the DMEM and FBS culture media. Any apoptosis and cellular loss for various reasons other than the cytotoxic effect of the sealers occurred in the control groups and ELX808 Absorbance Reader was calibrated at baseline based on the color change due to it (zero) and color changes of the other wells were determined based on it. The following formulas were used to determine the optical densities (OD) of the culture media with each sealer based on percentage cell viability relative to the control group: The absorbance (OD) of this colored solution was quantified by its measurement at a certain wavelength. By increased reduction of formazan and measurement of OD, cell viability and the cytotoxicity of materials can be measured: 

Cell Viability= (sample OD/control OD) ×100

Cytotoxicity=100-Cell Viability

Data provided by the ELISA reader were analyzed with descriptive statistics (means ± standard deviations); one-way ANOVA and repeated-measures ANOVA, using Statistical Package for Social Science (SPSS, version 20.0, SPSS, Chicago, IL, USA). Statistical significance was set at 0.05.

## Results

Tables 1 and 2 present the spectroscopy results of the samples in comparison with control data which is in relation to the metabolic activity of the cells. All sealers showed severe toxicity in all time intervals except for Apatite root canal sealer in 48-h time interval. One way ANOVA demonstrated significant differences in toxicity between the three sealers (*P*=0.001). In addition, Scheffé’s test showed that the toxicity of MTA Fillapex was significantly higher than that of Apatite root canal sealer (*P*=0.003). The toxicity of MTA Fillapex was not significantly different from that of AH-26 sealer (*P*=0.979). The toxicity of Apatite sealer was significantly less than that of AH-26 (*P*=0.002). At 48-h interval after mixing, ANOVA revealed significant differences in toxicity between the three sealers (*P*=0.000). In addition, Scheffé’s test showed that: The toxicity of MTA Fillapex was significantly higher than that of Apatite sealer (*P*=0.000). The toxicity of MTA Fillapex was not significantly different from that of AH-26 sealer (*P*=0.477). The toxicity of MTA Fillapex was significantly less than that of AH-26 sealer (*P*=0.000). At 72-h interval after mixing, ANOVA did not show any significant differences in toxicity between the three sealers (*P*=0.671). At 7-day interval after mixing, ANOVA did not reveal any significant differences in toxicity between the three sealers (*P*=0.488).

**Figure 1:**
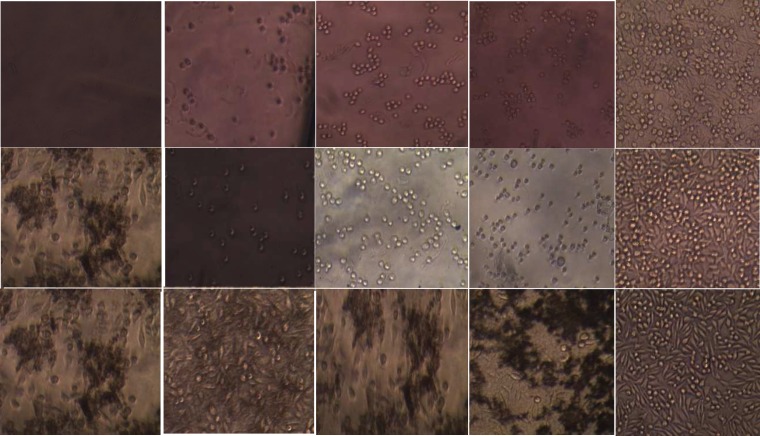
Light microscope image of the cells stained with the MTT technique in MTA Fillapex, AH-26 and Apatite root canal sealer and in the control group (400× magnification

**Figure 2:**
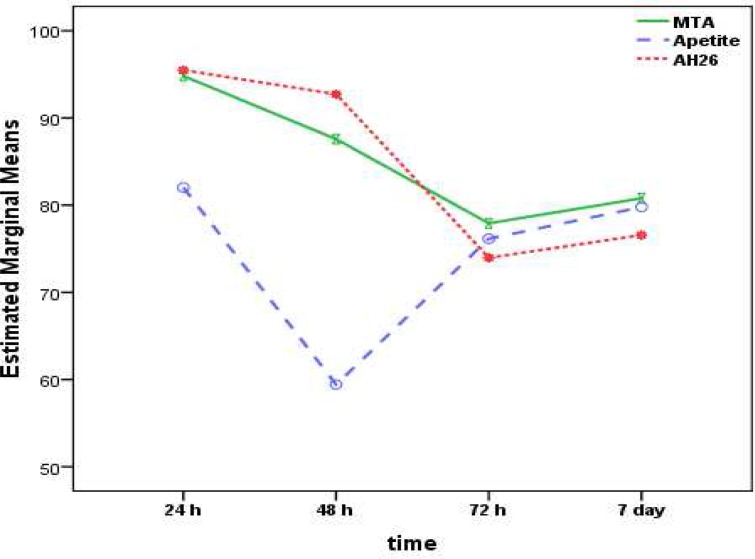
The ratio and consequent alterations in toxicity of MTA Fillapex, AH-26 and Apatite root canal sealer during the first 7 days

The results showed that there were changes in the toxicity of all the sealers over time. In this context, in MTA Fillapex and AH-26 sealers there was a decrease in toxicity over time; however, in the Apatite root canal sealer group there was a decrease in toxicity up to 48 h but toxicity increased from 48 to 72 h (Figures 1 and 2). 

## Discussion

Clinically, sealers are applied to the oral cavity freshly or incompletely polymerized after mixing; however, even after setting it is possible for potentially toxic agents to be released from these materials after their contact with tissue fluids [7]. It has been suggested that their cellular toxicity can be evaluated at different intervals using cell culture techniques [20]. 

The toxicity of the sealer depends on its concentration, the time lapse after mixing, the test type, the cell type used or the sealer being fresh or set [21]. Very different results have been reported in a large number of *in vitro* studies on sealers. Therefore, it is difficult or even impossible to compare the results because a large number of variables play a role in evaluation of their toxicity. 

MTT assay depends on the activity of succinate dehydrogenase and formation of insoluble blue crystals. Contrary to other techniques, the irrigation and harvesting procedures of cells that might result in the loss of a number of cells have been eliminated and all the steps of the assay from cell culturing to reading of the results by a photometer are carried out in a micro plate; therefore, the assay has high reproducibility, accuracy and sensitivity. Generally, cells in the culture media are found as attached to the plate or suspended. MTT assay is a spectrophotometry technique which depends on the reduction and breakage of yellow tetrazolium crystals with the chemical formula of 3-(4, 5-dimethylthiazolyl)-2, 5-diphenyltetrazolium bromide. This technique is easier than the other techniques used for the evaluation of cell proliferation and can be carried out with the facilities available in the majority of laboratories [22]. In addition, all test procedures are carried out in 96-well cell culture plates and the results are provided by an ELISA realer. Therefore, a large number of samples can be tested at the same time. The technique also has some disadvantages like lack of high sensitivity, chemical interference and some degree of toxicity [22]. Based on the protocol of this assay, if the assay is carried out on cells attached to the 96-well plate, such as tumoral cells and fibroblasts, first an adequate number of cells (preferably 5000 cells in each well) (9) should be cultured in each well. In the present study the HFFF2 cell line, was used. This cell line has been derived from a 14-18 week old human fetus and possess a finite life span. 

The results of the present study showed less cytotoxicity of Apatite sealer at all different time intervals and similar cytotoxicity of MTA Fillapex and AH-26. Also, the cytotoxicity of MTA Fillapex and AH-26 decreased after 48 h.

**Table 1:** Comparison of the cytotoxicity percentage (%) of MTA Fillapex, AH-26 and Apatite root canal sealer at different intervals

	**Mean (SD)**			
**Sealer **	**24 h**	**48 h**	**72 h**	**7 days**
**MTA**	94.81 (4.53)	87.57 (7.22)	77.92 (10.23)	80.79 (9.97)
**AH-26**	95.48 (5.95)	92.72 (5.26)	73.96 (3.92)	76.57 (2.58)
**Apatite**	82.02 (8.61)	59.42 (11.30)	76.14 (10.56)	79.78 (5.77)
***P*** ** value**	0.001	0.000	0.671	0.448

**Table 2:** Scheffé’s test for two-by-two comparison of the sealers in terms of toxicity at each time interval

	***P*** **-value**				
**(I)**	**(J)**	**24 h**	**48 h**	**72 h**	**7 days**
**MTA**	**Apatite**	0.003	0.000	0.922	0.957
**MTA**	**AH-26**	0.979	0.477	0.672	0.478
**Apatite**	**AH-26**	0.002	0.000	0.885	0.648

Assmann *et al.* [23] carried out a study on the osseous tissue response of MTA Fillapex and AH-Plus and concluded that MTA in the chemical composition of MTA Fillapex sealer had no effect on improving osseous tissue repair. The presence of sealers restored the original osseous tissue structure and the severity of inflammatory reaction decreased with time; therefore, these sealers were biocompatible.

Braga, *et al.* [24] assessed the cytotoxicity of MTA Fillapex sealer in relation to murine macrophage activity and reported that MTA Fillapex might interfere with macrophage activity even after 4 h of contact. In the present study, MTA Fillapex exhibited the maximum toxicity at 24- and 48-h intervals after mixing, which was statistically significant (*P*<0.05). The toxicity decreased significantly over time up to 72 h; however, there was no significant change in toxicity over time up to 7 days. Higher susceptibility of murine macrophage cells in comparison with foreskin fibroblasts may explain the difference in results. In addition, in the present study, MTA Fillapex induced a high rate of cellular necrosis from 24 h to 7 days. 

In a study by Yoshino *et al*. [20], the toxicity of MTA Fillapex was evaluated using MTT assay at 24-, 48- and 72-h intervals using periodontal ligament fibroblasts, and high toxicity of this sealer was reported. Bin *et al.* [21] conducted MTT assay on hamster fibroblasts and showed that MTA Fillapex sealer exhibited high cellular toxicity. Silva *et al.* [3], used MTT assay and reported that the toxicity of this sealer was very high initially and high toxicity remained to the end of the third week. The results of all these studies are consistent with those of the present study.

In addition, Chang *et al.* [2], concluded that none of the Sankin Apatite and MTA Fillapex root canal sealers were cytotoxic, contrary to the results of the present study, which might be attributed to the lower sensitivity of immortalized PDL cells compared to the cells evaluated in the present study. 

In the present study, AH-26 sealer, similar to MTA Fillapex sealer, exhibited the highest toxicity 24 and 48 h after mixing, which was statistically significant. However, the toxicity significantly decreased over time up to 72 h after mixing, with no significant changes in toxicity up to 7 days. Furthermore, from 24 h to 7 days, the toxicity of AH-26 was high (over 70% of cellular death), which might be attributed to the release of formaldehyde from this sealer, which is a toxic agent. 

Huang *et al. *[7], reported that AH-26 sealer exhibited high toxicity during the initial days, which decreased over time; however, its toxicity was still high, which is consistent with the results of the present study because in the present study, too, AH-26 exhibited high toxicity up to 48 h, which decreased over time. In addition, a study by Spangberg *et al. *[25], showed that AH-26 sealer exhibited the highest release of formaldehyde during the first 48 h, which decreased later. This finding can explain the results of the present study. 

Miletic *et al. *[16], reported that AH-26 was toxic at the beginning, without cytotoxic properties on the 7^th^ day after setting, contrary to the results of the present study; such a discrepancy in the results might be attributed to inclusion of V79 cells (pulmonary fibroblasts) in Chinese hamsters in the aforementioned study. 

In the present study, Apatite root canal sealer exhibited the minimum toxicity at 48-h interval; however, it exhibited no differences in toxicity at 24-h, 72-h and 7-day intervals. In other words, this sealer exhibited high toxicity on the day of mixing, which decreased to a minimum degree after 48 h. However, after 3 days toxicity increased significantly, with no changes in toxicity up to 7 days after mixing. This trend of changes in toxicity might be attributed to changes in the chemical formulation during the setting reaction and the release of various chemical agents during the setting reaction, necessitating more accurate evaluations by designing further studies to determine the chemical formulation of the sealer at different intervals after mixing. 

Kim *et al.* [26], showed that the toxicity of first and third generations of Apatite root canal sealer was less than that of AH-26 and more than that of ZOE-based sealers. In the present study, too, the toxicity of Apatite sealer was less than AH-26. The lower toxicity of hydroxyapatite might be attributed to the fact that this sealer is an important biomaterial and is the main inorganic consistent of bone and tooth, resulting in excellent biocompatibility of this sealer. In addition, it exhibits very low solubility due to its low porosity and high density [27], resulting in its low toxicity. 

Finally, it should be pointed out that this study evaluated the cellular toxicity at the mentioned time intervals and further studies are necessary for the evaluation of reasons for and interpretation of collected data.

## Conclusion

All the three sealers, AH-26, MTA Fillapex and Apatite, were toxic during the 7 days of *in vitro *evaluation. Toxicity was significantly high initially and gradually decreased over time. Apatite root canal sealer exhibited the minimum toxicity. AH-26 and MTA Fillapex exhibited similar toxicity; however, both were more toxic than Apatite root canal sealer.
